# The Effects of Sodium Antimonate on the Flame Retardancy and Mechanical Properties of Thermoplastic Polyurethane Composites

**DOI:** 10.3390/molecules31183200

**Published:** 2026-09-10

**Authors:** Xinchao Wang, Shaobin Cai, Chenhao Xu, Tie Geng, Xiaoli Bai, Jiayu Liao, Tongfei Zhang, Baichuan He, Pengyu He, Mengling Li

**Affiliations:** National Engineering Research Center of Wheat and Corn Further Processing, School of Mechanical and Electrical Engineering, Henan University of Technology, No. 100, Lianhua Street, National High-Tech Development Zone, Zhengzhou 450001, China; xinchaowang2016@haut.edu.cn (X.W.); 3395455232@qq.com (S.C.); 2428281629@qq.com (C.X.); 3312769628@qq.com (J.L.); 3031566647@qq.com (T.Z.); 2093549406@qq.com (B.H.); hpy866@qq.com (P.H.); lixiaojie_69@qq.com (M.L.)

**Keywords:** sodium antimonate, thermoplastic polyurethane elastomer (TPU), flame-retardant properties, thermal stability, mechanical properties, flame-retardant mechanism

## Abstract

Thermoplastic polyurethane (TPU) is highly versatile yet inherently flammable, restricting its use in fire-safe applications. This study incorporates sodium antimonate (SA) into TPU via melt blending (0–10 wt%). Through a comprehensive suite of analytical techniques—including thermogravimetric analysis (TGA), Fourier transform infrared spectroscopy (FT-IR), universal testing, and cone calorimetry—the effects of SA on the various properties of the material were investigated. Quantitative analysis reveals that 10 wt% SA delivers the best flame retardancy, reducing the peak heat release rate by ~50% (from 605 to 310 kW/m^2^) and total heat release by ~40% (from 32 to 19 MJ/m^2^), while increasing the char residue from 9.84% to 13.89%. However, to retain mechanical robustness, the SA content must be capped at ≤5 wt%. This ensures that the material retains 30% of its fracture elongation and a tensile strength of 1.7 MPa; higher loadings cause severe embrittlement due to particle agglomeration. Mechanistically, SA acts via a dual-phase mode—promoting a dense insulating char layer in the condensed phase and quenching reactive radicals in the gas phase. These findings establish a practical balance between fire safety and mechanical performance, offering a clear guideline for the rational design of flame-retardant TPU composites.

## 1. Introduction

Thermoplastic polyurethane (TPU) is widely used in the automotive, electronics, and construction sectors due to its outstanding elasticity and processability [1,2]. However, its intrinsic flammability (LOI~18–20%), accompanied by heavy smoke and melt dripping, severely limits compliance with fire-safety standards such as UL 94 V-0 and low heat-release requirements in transportation [3,4,5,6]. The hydrogen halide gases generated by traditional halogenated flame retardants during combustion are highly corrosive and toxic [7,8]; consequently, their use is gradually being restricted. SA (NaSbO_3_), as a typical inorganic flame retardant synergist, can promote char layer formation and inhibit combustion reactions [9,10,11]. SA is characterized by low addition levels and high flame-retardant efficiency, demonstrating promising application potential in the field of flame retardation for polymeric materials. In recent years, TPU flame retardant systems have primarily focused on phosphorus-based, nitrogen-based, inorganic, and composite systems [12,13,14]. Phosphorus-based alternatives require high loadings (>20 wt%) that plasticize and weaken TPU [15], while nitrogen-based flame retardants are often used synergistically with other flame retardants [16,17]. When inorganic flame retardants act synergistically with other flame retardants, they can significantly reduce smoke density [18].

When used synergistically with halogenated flame retardants, SA can significantly enhance the flame retardancy efficiency of materials [19,20]. Existing studies indicate that Sb_2_O_3_—generated from the high-temperature decomposition of antimony-based compounds—catalyzes the carbonization of polymers, forming a dense carbonaceous barrier that terminates chain reactions [7,21,22,23]. Inorganic acid salts can achieve enhanced flame retardancy by promoting char formation and reducing the heat release rate and smoke emission [1,7,18,21,22]. The LOI value of the TPU-SA10 group reached 32.8%, and it achieved a V-0 rating in the vertical burning test. Furthermore, the pHRR and THR were reduced by 50% and 40%, respectively, compared to pure TPU, resulting in a significant reduction in fire hazard. Despite these known benefits, the standalone application of SA in thermoplastic polyurethane elastomers—a polymer with a delicate microphase-separated structure—remains scarcely investigated. Critically, three unresolved issues persist: (i) how SA particles affect the hydrogen-bonding network and soft/hard segment dynamics that govern TPU’s resilience, (ii) whether SA can effectively catalyze charring of the urethane soft segments under real fire conditions, and (iii) the quantitative loading threshold beyond which flame-retardant gains are offset by mechanical embrittlement.

To address this gap, the present work prepares SA/TPU composites across 0–10 wt% via melt blending. Through systematic TGA, FT-IR, cone calorimetry, and tensile testing, we aim to (1) quantify the improvements in thermal stability and fire performance; (2) identify the optimal SA loading that preserves part of the original mechanical properties while significantly reducing fire hazard (e.g., ~50% reduction in peak heat release); and (3) elucidate the dual-phase flame-retardant mechanism. The novelty lies in establishing the first quantitative balance guideline for SA/TPU, providing actionable data for fire-safe formulations in high-risk applications such as electric vehicle battery enclosures, flexible electronic substrates, and fire-retardant conveyor belts, where both safety and mechanical robustness are imperative.

## 2. Results and Discussion

### 2.1. Effect of SA on the Thermal Stability of TPU

**Figure 1:**
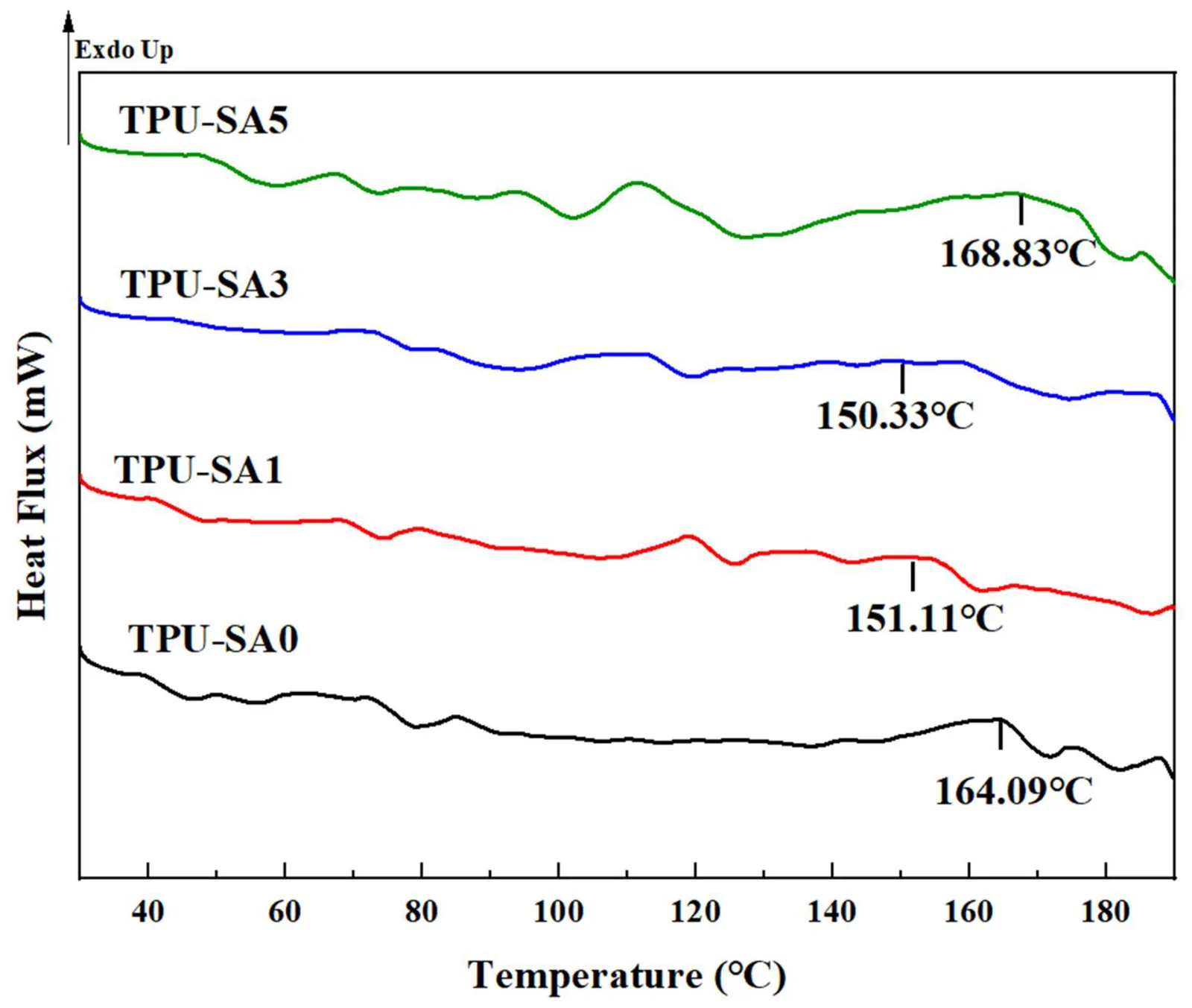
DSC test curve.

Figure 1 presents the DSC curves for pure TPU (TPU-SA0) and composite systems containing varying amounts of SA (TPU-SA1, TPU-SA3, and TPU-SA5). As shown in the figure, pure TPU exhibits a glass transition step associated with the soft segments in the range of 60–70 °C, followed by an endothermic melting peak corresponding to the hard segments between 150 °C and 170 °C; this behavior is consistent with the typical thermal characteristics of TPU. Upon the addition of SA, the glass transition temperature of the composite systems shifts gradually toward higher temperatures as the additive content increases. This indicates that the SA establishes interfacial interactions with the TPU molecular chains, thereby restricting the molecular motion of the soft segments and enhancing the rigidity of the matrix. Simultaneously, the melting peaks of the hard segments progressively diminish in intensity and broaden in shape, suggesting that the SA inhibits the ordered crystallization of the TPU hard segments and effectively modulates the aggregated structure of the matrix [24]. All samples exhibit a single glass transition event devoid of any additional phase transition peaks, thereby confirming that the SA is uniformly dispersed within the TPU matrix and demonstrates excellent compatibility. This structural foundation serves as the basis for the synergistic enhancement of both the mechanical properties and flame retardancy of the composite systems [25]. Furthermore, minor baseline fluctuations observed in the DSC curves are attributed to the microphase-separated morphology of TPU and the dispersion of inorganic SA particles; however, the thermal transition temperatures are reproducible and clearly identifiable.

**Figure 2:**
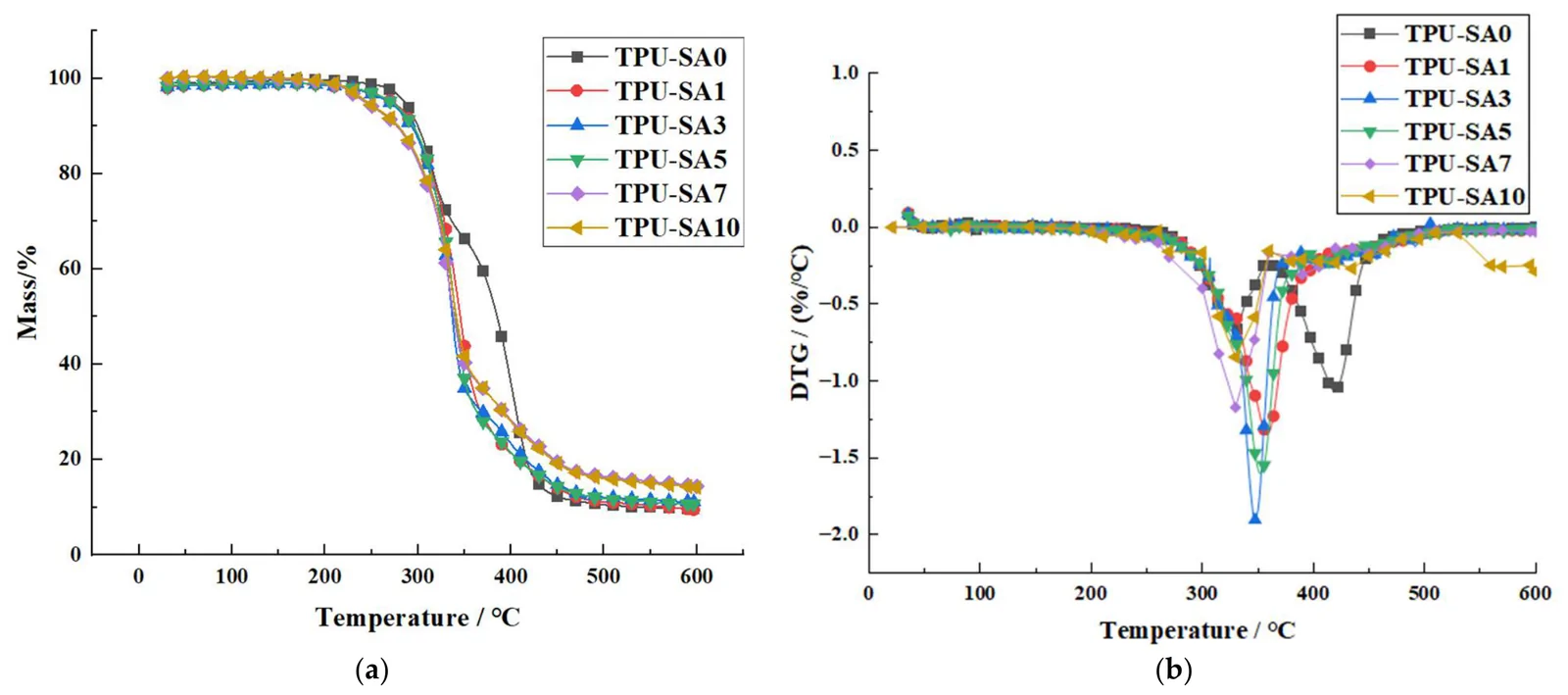
(**a**) Thermogravimetric analysis (TGA) and (**b**) the derivative curves (DTG).

Figure 2 presents the TGA thermogravimetric curves, the derivative curves (DTG), and characteristic thermal decomposition parameters for the SA/TPU composite system. Table 1 lists the T_5%_ (temperature at which the mass loss is 5%), T_50%_ (temperature at which the mass loss is 50%), maximum thermal decomposition temperature (T_max_), and residual carbon content for each sample with different compositions. As shown in the figure, the pure TPU-SA0 exhibits an initial thermal decomposition temperature of 301.84 °C, a maximum thermal decomposition rate temperature of 419.5 °C, and a residual carbon yield of only 9.84% at 600 °C, indicating relatively poor thermal stability [26,27]. At lower loadings (≤3 wt%), SA decomposes prematurely, releasing antimony species (e.g., Sb_2_O_3_) that act as Lewis acids, catalyzing the scission of urethane bonds. This catalytic effect reduces both T_5%_ and T_max_, as reflected in Table 1. At higher loadings (≥7 wt%), the accumulated antimony oxides promote early cross-linking and char formation. The char layer acts as a physical barrier that suppresses heat/mass transfer, partially offsetting the catalytic degradation, as evidenced by the flattened DTG peak and increased char residue. This “catalytic promotion vs. barrier inhibition” competition provides a comprehensive explanation for the non-monotonic trend in T_max_ and the progressive increase in residue yield with SA content. Upon the addition of SA, the thermal decomposition behavior of the composite system undergoes significant changes: as the loading of SA increases, the initial thermal decomposition temperature decreases slightly—a phenomenon attributed to the premature decomposition of SA triggering the catalytic degradation of TPU. Concurrently, the crest value corresponding to the maximum thermal decomposition rate decreases significantly, indicating that SA effectively retards the thermal decomposition rate of TPU; for pure TPU, the main decomposition in TGA/DTG exhibits a broad temperature range and a flat peak, indicating stepwise pyrolysis of soft and hard segments. Low-sodium antimonate loading exerts strong catalytic cracking, accelerating chain scission and shifting the decomposition to lower temperatures with a sharp, narrow DTG peak and earlier T_max_. Higher loading produces a continuous, dense antimony-containing char layer, which acts as a physical barrier to suppress heat/mass transfer and retard further cracking. Consequently, the decomposition zone slightly widens, T_max_ recovers, and the DTG peak flattens, revealing a dual role of catalytic promotion and barrier inhibition, thereby preventing concentrated heat release [28,29,30]. Furthermore, the residual carbon yield increases substantially; for sample TPU-SA7, the residual carbon yield reaches 13.89%, representing a significant improvement over that of pure TPU [31,32]. This enhancement is attributed to the antimony oxides generated from the decomposition of SA, which catalyze the cross-linking of TPU molecular chains into char. This process reduces the generation of volatile products, thereby effectively enhancing the material’s thermal stability and laying a solid foundation for improved flame retardancy.

### 2.2. Analysis of Infrared Spectroscopy Characterization Results

**Figure 3:**
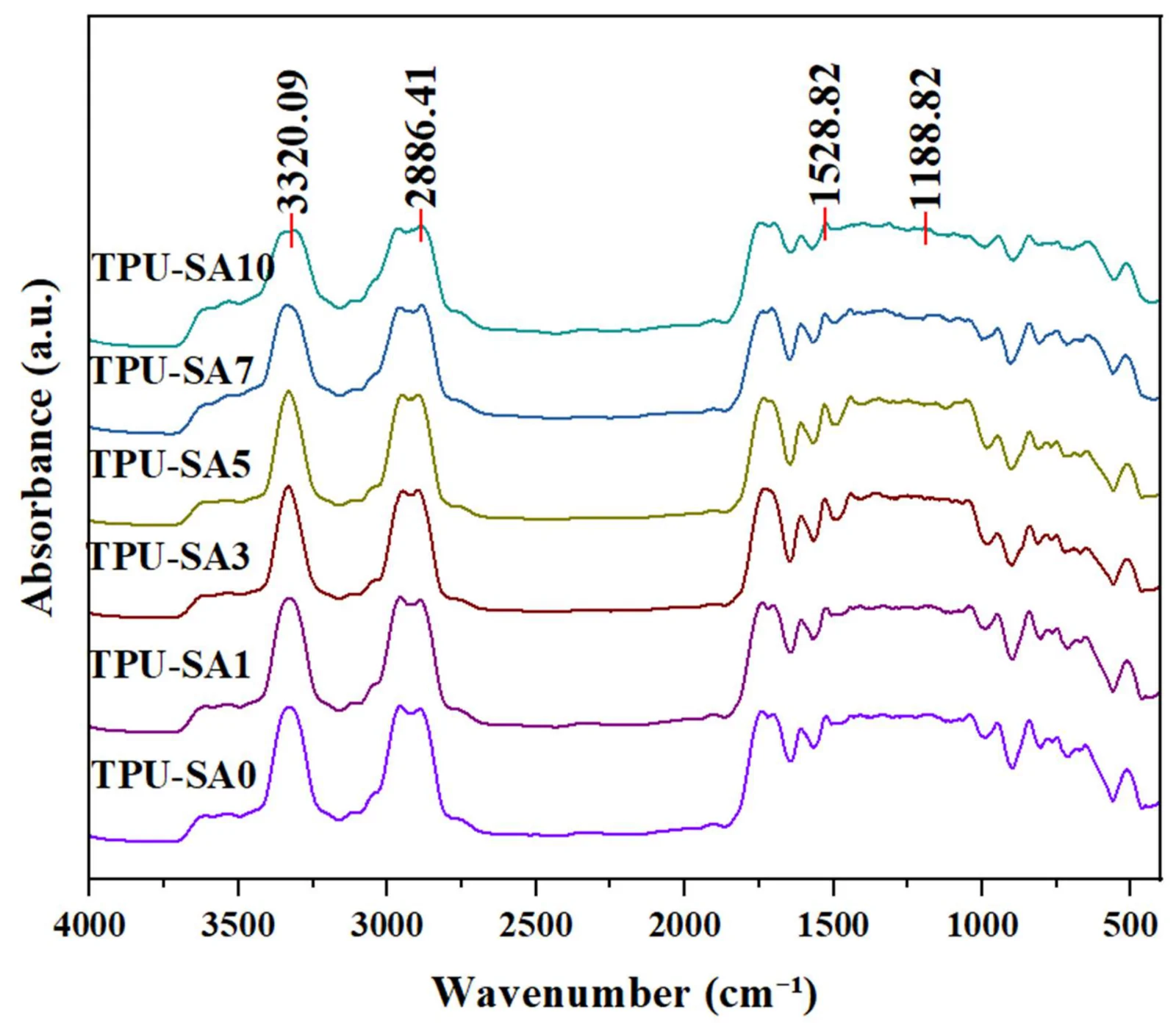
Room-temperature FTIR spectra of composite materials with different formulations.

As shown in Figure 3, room-temperature FTIR spectra of TPU composites with varying SA contents (0–10 wt%) were analyzed. Pure TPU-SA0 shows characteristic peaks: N-H stretching (3320.09 cm^−1^), -CH_2_-stretching (2886.41 cm^−1^), N-H bending/C-N coupling (1528.82 cm^−1^), and C-O-C stretching (1188.82 cm^−1^), confirming typical soft/hard segment structure. The N-H stretching band shifts from 3320.09 cm^−1^ (pure TPU) to 3315.26 cm^−1^ (TPU-SA10), representing a ~4.8 cm^−1^ red shift. Additionally, the C=O band shifts from 1703.12 cm^−1^ to 1700.85 cm^−1^ (a ~2.3 cm^−1^ shift). No new peak or peak disappearance is observed upon filler addition, indicating no chemical reaction or covalent bonding. However, the N-H stretching peak gradually shifts to lower wavenumbers and broadens with increasing filler content, revealing intermolecular hydrogen bonding between SA (oxygen atoms as acceptors) and TPU’s N-H groups. This enhances interfacial compatibility and chain interactions, aiding structural stability.

**Figure 4:**
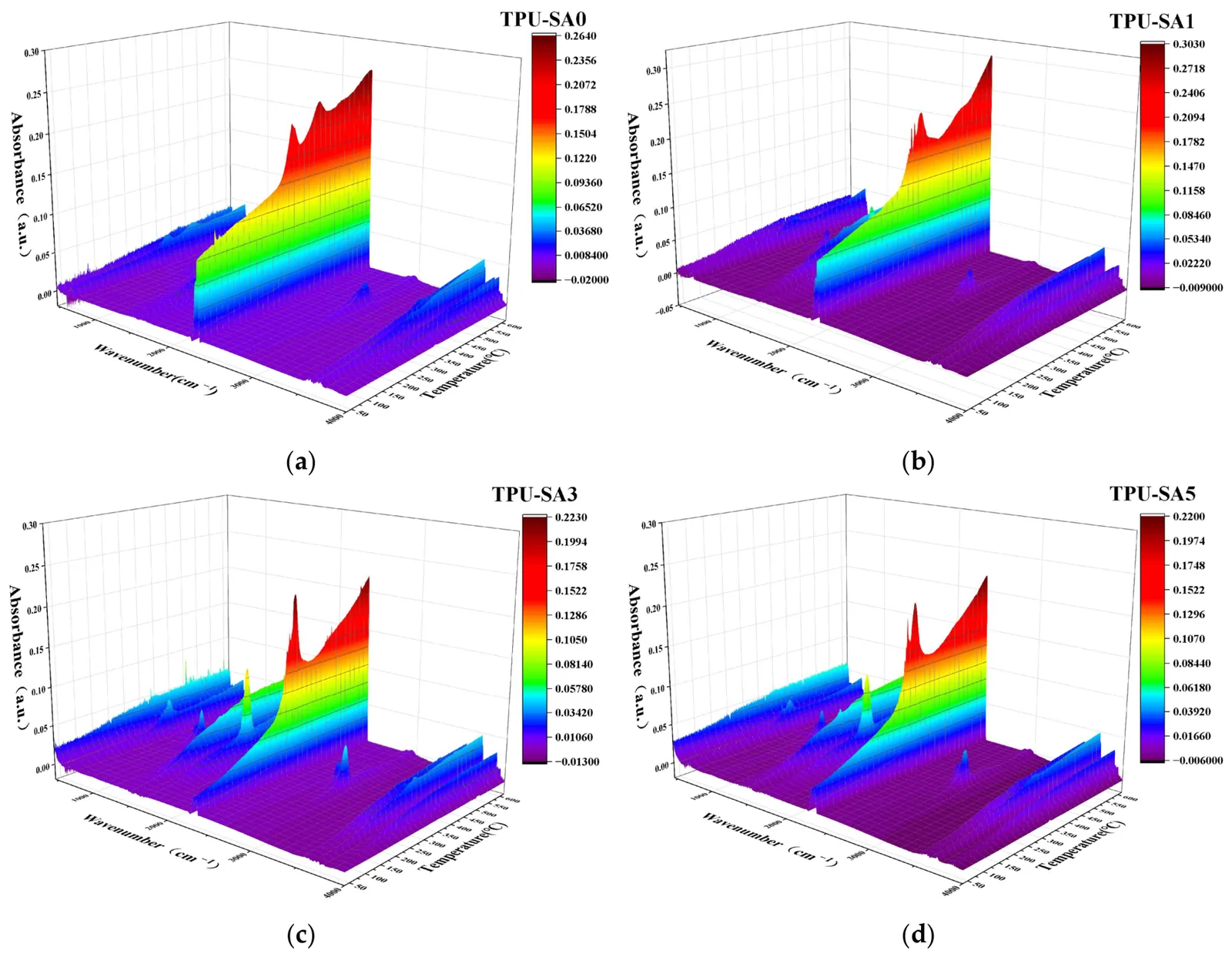
The 3D variable-temperature infrared spectra of (**a**) TPU + 0% SA, (**b**) TPU + 1% SA, (**c**) TPU + 3% SA, and (**d**) TPU + 5% SA.

Figure 4 presents the 3D temperature-dependent infrared spectra of pure TPU and composite systems containing varying amounts of SA composites. As illustrated in the figure, TPU-SA0 exhibits distinct characteristic urethane peaks (e.g., N-H at 3326 cm^−1^ and C=O at 1703 cm^−1^) in the low-temperature range; however, upon heating, these characteristic peaks decay rapidly, leaving virtually no residual signals in the high-temperature range, indicating complete thermal decomposition [33,34]. With the addition of SA, the infrared spectra of the composite systems undergo significant changes: in the low-temperature range, the intensities of the N-H and C=O characteristic peaks decrease and shift toward lower wavenumbers, indicating the formation of hydrogen-bonding interactions between the SA and the TPU molecular chains, thereby enhancing interfacial compatibility [22,33]. In the medium-temperature range, the decay rate of the characteristic peaks is markedly retarded, demonstrating that SA effectively inhibits the thermal decomposition of the TPU molecular chains [35]. Furthermore, in the high-temperature range, the intensity of the aromatic C=C peak—located near 1600 cm^−1^—increases significantly as the SA content rises; this confirms that the antimony oxides generated from the decomposition of SA catalyze the aromatization of the TPU molecular chains, thereby promoting the formation of a stable aromatic char layer. This finding aligns with the increased residual carbon yields observed in the TGA results, thereby elucidating—at the molecular level—the condensed-phase char-forming flame-retardant mechanism of SA. The surface of SA particles contains oxygen-rich antimonate groups (Sb-O-Sb and Sb-O-Na), where the oxygen atoms act as hydrogen-bond acceptors. These interact with the N-H proton donors of TPU’s urethane linkages, forming hydrogen bonds. This interaction enhances interfacial compatibility and restricts chain mobility, consistent with the increased T_g_ observed in DSC.

### 2.3. Effect of SA on the Mechanical Properties of TPU

**Figure 5:**
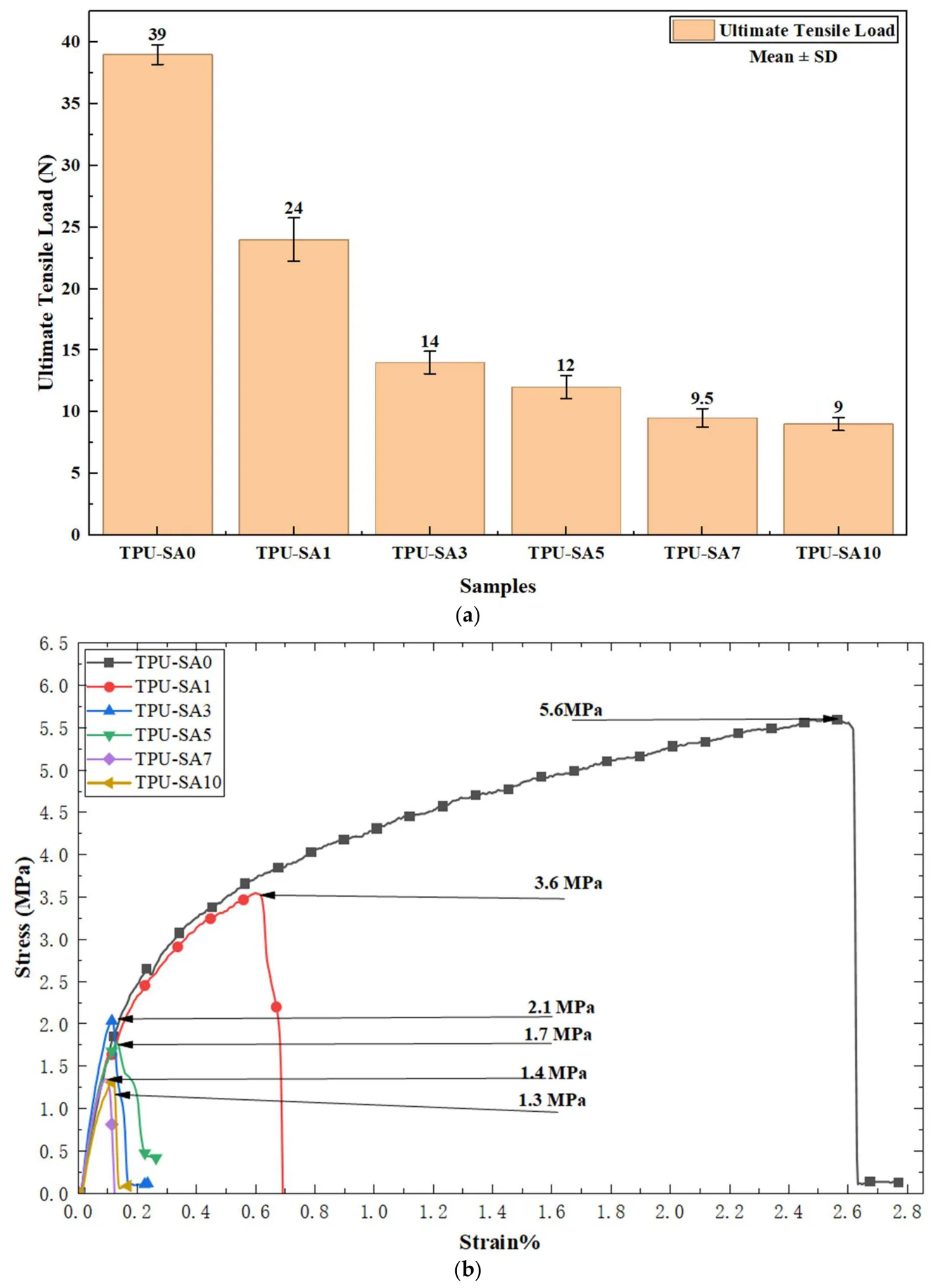
Influence curves on mechanical properties of (**a**) ultimate tensile load and (**b**) stress–strain.

The mechanical properties of TPU materials serve as a crucial guarantee for their practical applications; as an inorganic filler, the loading level and dispersion of SA directly influence the mechanical properties of the composite system. Figure 5 presents the stress-strain curves, maximum stress variation curves, and a bar chart of the maximum tensile force for composite systems containing varying amounts of SA.

Table 2 illustrates the simultaneous decrease in tensile strength and elongation at break as the SA content increases, indicating a transition of the material from ductile to brittle behavior; this reflects the trade-off between improved thermal stability and flame retardancy versus the loss of mechanical properties in the SA fulminate-modified TPU system. The tensile toughness data were derived from the integral of the stress–strain curve. The slight increase in the elongation at break of TPU-SA5 demonstrates that, within a certain threshold, SA can exert a reinforcing effect that enhances the elongation at break. Overall, at low modification levels, the material retains certain load-bearing capacity and plasticity characteristics; however, at high modification levels, the mechanical properties undergo significant degradation, making it unsuitable for conventional structural applications.

As evident from the figure, TPU-SA0 exhibits a maximum tensile stress of approximately 5.6 MPa, a maximum tensile force of 39 N, and a strain at break approaching 2.8, demonstrating excellent mechanical properties and high toughness. Upon the addition of SA, the mechanical properties of the composite system display a continuous downward trend:

Low-addition group (TPU-SA0, TPU-SA1): The maximum stress decreases to approximately 3.6 MPa, the maximum tensile force to 24 N, and the strain at break shortens to around 0.6; while these values represent a decline compared to the TPU-SA0 group, the material still retains a certain degree of toughness.

Medium-addition group (TPU-SA03, TPU-SA05): The maximum stress drops to 2.1 MPa and 1.7 MPa, respectively, and the maximum tensile force falls to 14 N and 12 N; the strain at break shortens further, and toughness declines significantly.

High-addition group (TPU-SA7, TPU-SA10): The maximum stress stands at merely 1.4 MPa and 1.3 MPa, and the maximum tensile force is 9.5 N and 9 N; the strain at break is extremely low, and the material undergoes distinct brittle fracture.

The fundamental reason for this change is that SA, acting as a rigid inorganic filler, disrupts the continuous-phase structure of the TPU matrix. When added in appropriate amounts, the filler disperses relatively uniformly, and its impact on mechanical properties remains relatively controllable. However, when the addition level exceeds 5%, the SA particles tend to agglomerate, creating defects and stress concentration points within the matrix; an abrupt drop in tensile properties beyond 5 wt%; and a non-monotonic trend in elastic modulus (increasing up to 3 wt% then decreasing at 10 wt%), which is characteristic of filler agglomeration-induced embrittlement and well-established literature precedents on inorganic fillers in TPU composites. This renders the material prone to fracture during tensile testing, resulting in a significant decline in both load-bearing capacity and toughness.

Overall, the incorporation of SA leads to a significant reduction in the mechanical properties of TPU; furthermore, the lower the additive content, the less pronounced the impact on these mechanical properties. If it is necessary to balance both flame retardancy and mechanical performance, it is recommended to limit the addition of SA to within 5%. At this level, the composite system retains a certain degree of mechanical integrity, thereby achieving a relative equilibrium between flame retardancy and mechanical properties that satisfies basic application requirements.

### 2.4. Effect of SA on the Combustion Performance of TPU

#### 2.4.1. Cone Calorimeter Test Results

**Figure 6:**
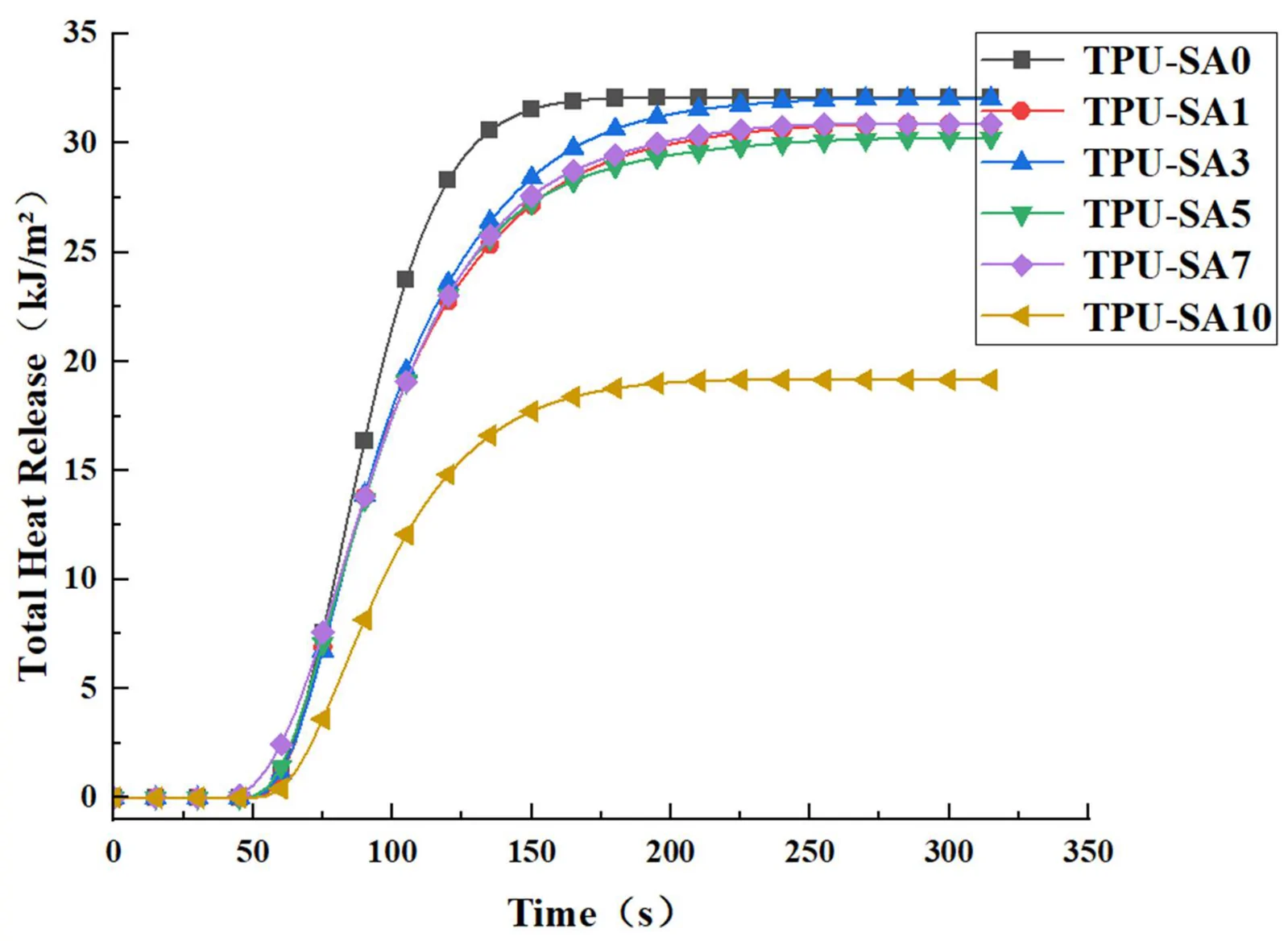
Total heat release curve.

Figure 6 presents the total heat release (THR) curves for TPU composite systems containing varying amounts of SA. Total heat release is a critical metric for assessing the fire hazard potential of a material; higher values indicate that the material releases a greater total amount of heat during combustion, thereby leading to more rapid fire propagation and more severe damage. As illustrated in the figure, TPU-SA0 exhibits an extremely rapid rate of heat release during the initial stages of combustion, reaching a final THR value of 32 MJ/m^2^, which signifies a very high fire hazard. Upon the addition of SA, the slope of the THR curve for the composite system decreases significantly; furthermore, as the additive content increases, the final saturation value continues to decline. Among the tested samples, the TPU-SA10 group demonstrates the most superior performance, with its final THR value dropping to 19 MJ/m^2^, a reduction of nearly 40% compared to pure TPU. This indicates that the incorporation of SA effectively inhibits the cumulative release of heat during the TPU combustion process, thereby substantially mitigating the material’s fire hazard. The fundamental mechanism underlying this effect is that the antimony oxides generated from the high-temperature decomposition of SA catalyze the formation of a dense char layer on the TPU surface; this layer acts as a barrier to heat transfer, thereby fundamentally reducing the generation and release of combustion products.

**Figure 7:**
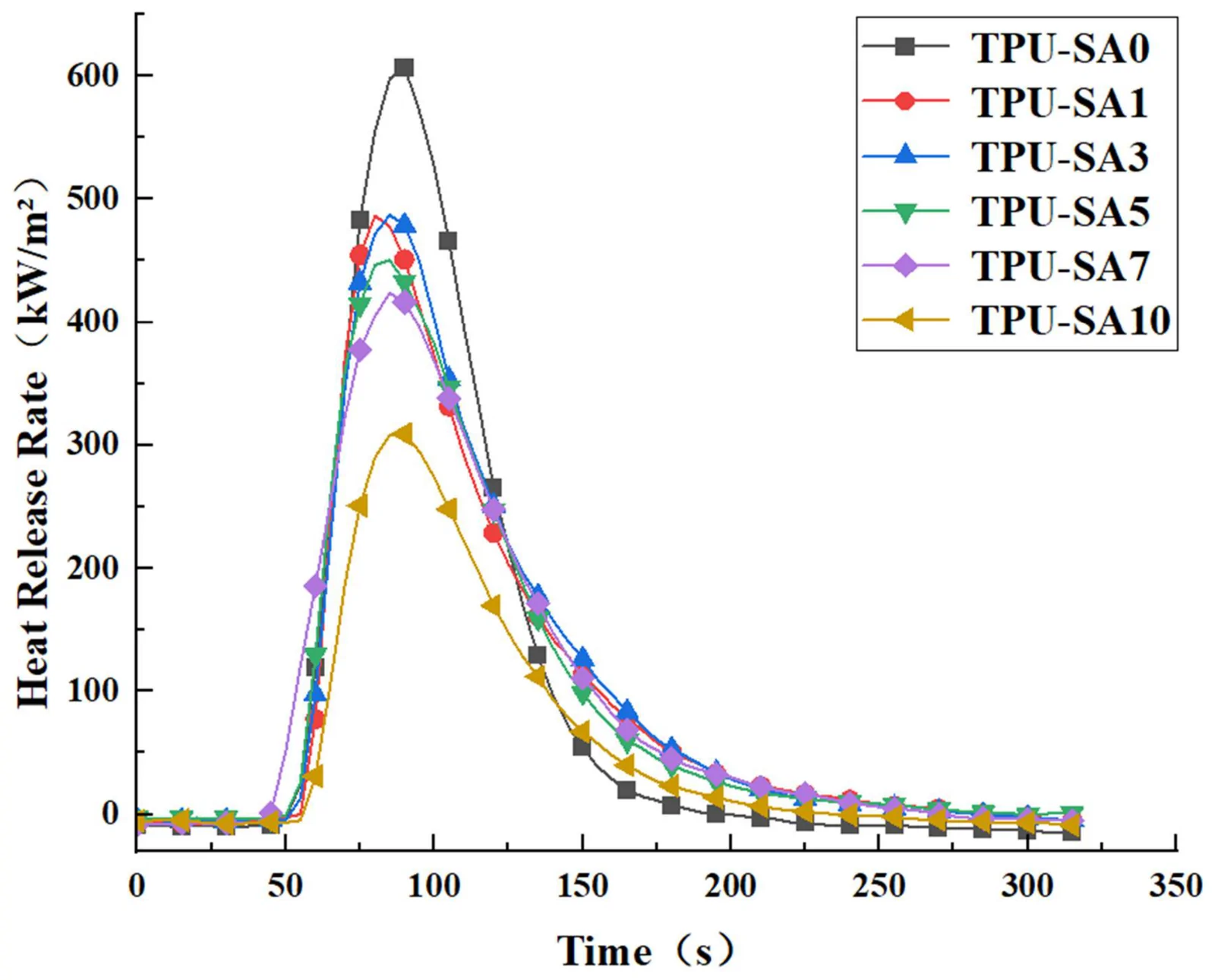
Heat release rate curve.

Figure 7 presents the heat release rate (HRR) curves for TPU composite systems containing varying amounts of SA. The heat release rate serves as a critical metric for assessing the intensity of material combustion and its associated fire hazard; a higher peak value and a sharper peak profile indicate more intense combustion and a greater risk of flashover [36,37]. As illustrated in the figure, the peak heat release rate of TPU-SA0 reaches as high as 605 kW/m^2^, characterized by a sharp peak profile; its combustion is extremely intense, posing a significant fire hazard. Upon the addition of SA, the peak HRR of the composite system decreases significantly as the additive content increases, while the peak profile becomes noticeably broader: for the TPU-SA10 group, the peak heat release rate drops to 310 kW/m^2^—a reduction of nearly 50% compared to pure TPU—indicating a significantly attenuated combustion process and a substantially reduced risk of flashover. This demonstrates that SA effectively suppresses the combustion intensity of TPU; its underlying mechanism involves catalyzing the formation of a dense char layer on the TPU surface at high temperatures—thereby blocking the transfer of heat and oxygen—while simultaneously scavenging active free radicals in the gas phase to terminate chain reactions, thereby achieving highly efficient flame retardancy.

#### 2.4.2. Analysis of the Microscopic Morphology of the Carbon Layer

**Figure 8:**
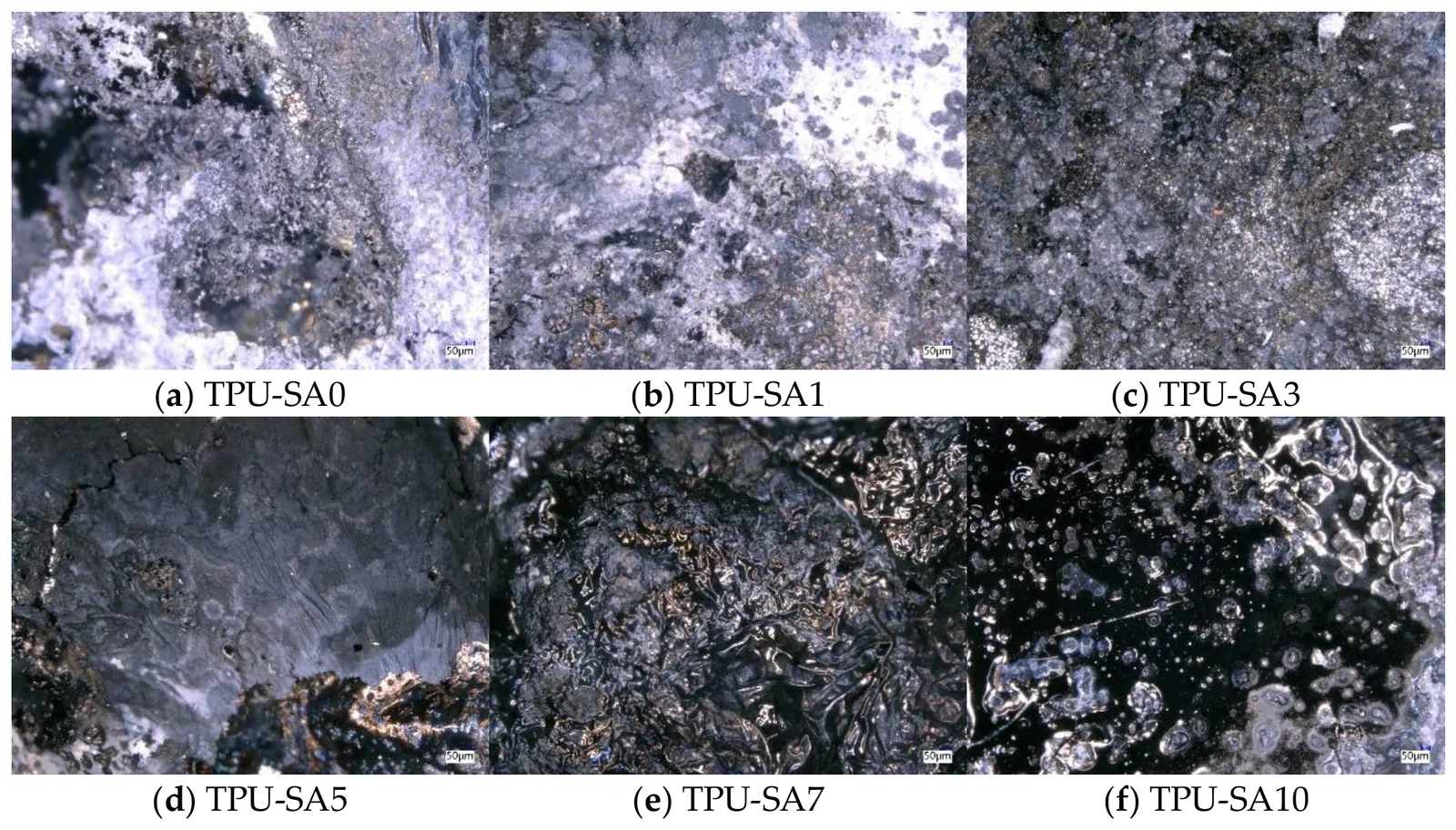
Ultra-depth-of-field morphology of combustion residues from different samples (100×).

The structure and morphology of the char layer formed after combustion serve as crucial evidence for elucidating flame-retardant mechanisms; Figure 8 presents SEM images of the char layers of various samples after combustion. Following combustion, TPU-SA0 formed almost no intact char layer, leaving behind only a small amount of loose, flocculent residue; its surface was riddled with pores and cracks, rendering it incapable of acting as a barrier against heat and oxygen (Figure 8a).

Upon the addition of SA, the structure of the char layer formed by the composite system after combustion showed gradual improvement: the char layer of the TPU-SA3 sample began to exhibit a continuous structure, though it still contained numerous micropores and cracks, resulting in poor compactness (Figure 8c); for the TPU-SA5 sample, porosity in the char layer decreased and the structure tended toward greater compactness, forming a rudimentary continuous char layer (Figure 8d); the compactness of the char layer in the TPU-SA7 sample improved significantly, presenting a relatively smooth surface with only a few minute pores (Figure 8e); finally, the char layer of the TPU-SA10 sample displayed a dense, continuous network structure devoid of obvious cracks or large pores (Figure 8f). Such a dense char layer effectively impedes heat transfer and oxygen diffusion, thereby inhibiting the further combustion of the TPU matrix while simultaneously reducing the release of smoke and volatile products, ultimately achieving the dual effects of flame retardation and smoke suppression. The changes in char layer morphology are consistent with the results of the preceding thermal stability and combustion performance tests, demonstrating that SA enhances the flame retardancy of TPU by promoting the formation of a dense char layer [19,38,39].

### 2.5. Flame Retardancy Mechanism of SP/TPU Composites

Based on the aforementioned results regarding thermal stability, mechanical properties, combustion behavior, and structural characterization, the flame-retardant mechanism of SA within the TPU system can be summarized as a synergistic effect involving both condensed-phase and gas-phase flame retardation.

**Figure 9:**
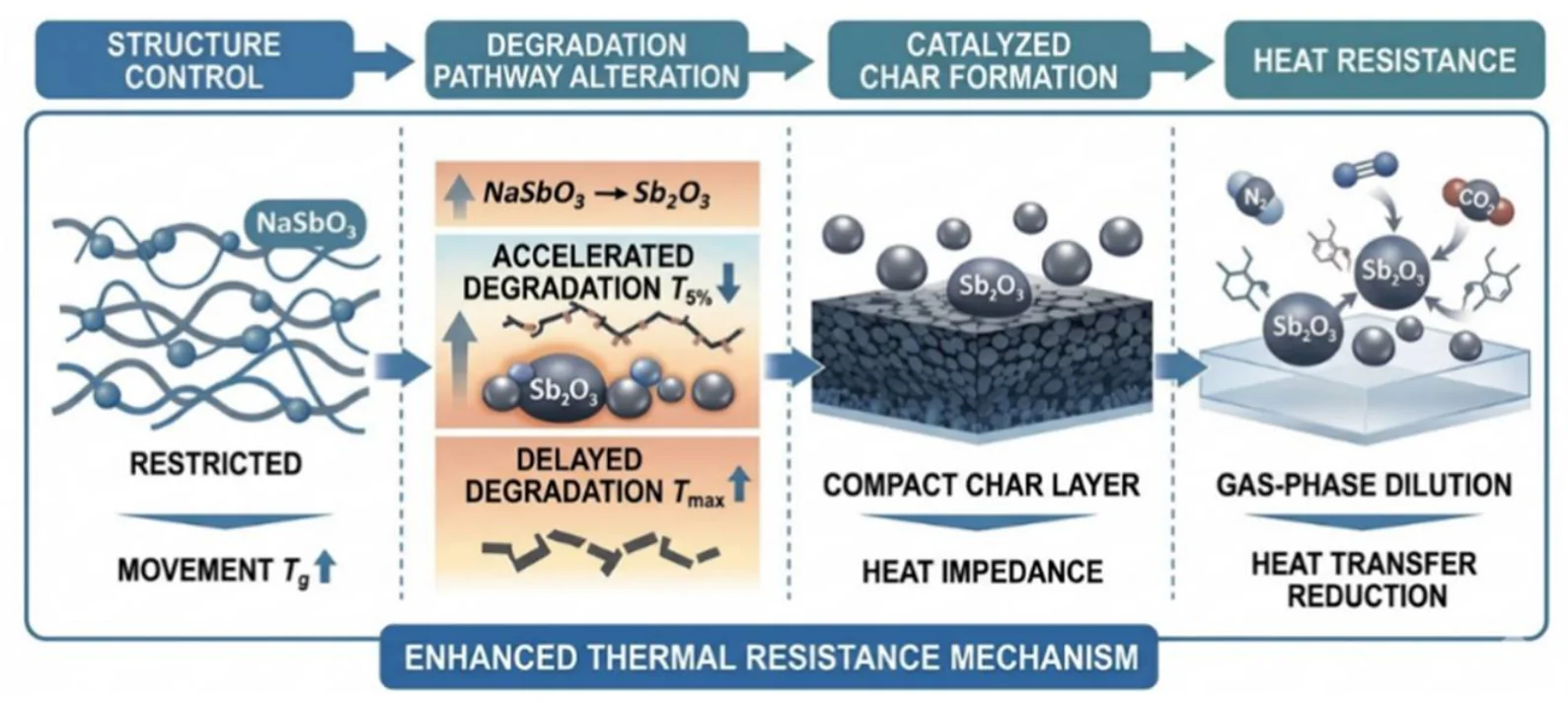
Flame retardancy mechanism diagram.

Condensed-phase flame-retardant mechanism (Figure 9): At high temperatures, SA decomposes to generate antimony oxides—such as Sb_2_O_3_—which act as Lewis acid catalysts; these oxides catalyze cross-linking and aromatization reactions within the TPU molecular chains, thereby promoting the formation of a dense, continuous char layer. This char layer exhibits excellent thermal stability and barrier properties; on one hand, it impedes the transfer of heat into the interior of the TPU matrix, thereby delaying the thermal decomposition of the matrix; on the other hand, it isolates the matrix from contact with oxygen, thereby cutting off the oxygen supply to the combustion reaction. Simultaneously, the dense char layer also suppresses the dripping of molten TPU and reduces the dispersion of burning materials, thereby inhibiting the spread of fire. Furthermore, the inorganic residues generated from the decomposition of SA can fill the pores within the char layer, further enhancing the density and stability of the char layer and reinforcing its barrier effect [18,40,41].

Gas-phase flame retardancy mechanism: During the decomposition of SA, active free radicals—such as SbO—are released. Within the gas-phase combustion zone, these radicals capture other active free radicals generated by the combustion reaction—such as HO· and H—thereby terminating the free-radical chain reactions, reducing the rate of gas-phase combustion, and consequently minimizing the release of heat and smoke. Simultaneously, the non-combustible gases generated by the decomposition of SA (such as Na_2_O-related products) can dilute the concentration of combustible gases within the combustion zone, thereby reducing combustion intensity and further enhancing the flame-retardant effect.

Through the synergistic action of two flame-retardant mechanisms, SA significantly enhances the thermal stability and flame-retardant properties of TPU; when added in appropriate amounts, it preserves the excellent mechanical properties of TPU, thereby achieving a balance between flame retardancy and mechanical performance.

## 3. Materials and Methods

### 3.1. Experimental Raw Materials and Reagents

The raw materials and reagents used in the experiments included thermoplastic polyurethane (TPU), grade TPU-1180A, density: 1.11 g/cm^3^ (ASTM D792 [42]); melt flow rate: 12 g/10 min (190 °C, 8.7 kg; ASTM D1238 [43]), serving as the composite matrix; SA (NaSbO_3_), with a purity of no less than 99.5%, a particle size not exceeding 5 μm, and a whiteness of no less than 92%, used as an inorganic flame-retardant functional filler; Antioxidant 1010 (a hindered phenol), with a purity of no less than 98%, selected to inhibit TPU degradation during processing and testing; and stearamide, with a purity of no less than 98%, serving as a dispersant to improve the dispersion of SA within the TPU matrix.

### 3.2. Experimental Instruments and Equipment

The experimental instruments and equipment utilized included a vacuum drying oven (Model DZF-6050, Shanghai Yiheng Scientific Instrument Co., Ltd., Shanghai, China) for drying raw materials and removing moisture; a high-speed mixer (SHR-10A, Zhangjiagang Huachang Machinery Co., Ltd., Zhangjiagang, China) for the premixing of raw materials; a twin-screw extruder (TE-35, Nanjing Kechuang Extrusion Equipment Co., Ltd., Nanjing, China) for the melt blending and granulation of composite materials; an injection molding machine (HTF86X1, Haitian Plastic Machinery Co., Ltd., Ningbo, China) for the preparation of standard test specimens; a four-point probe tester (RTS-9, Guangzhou Four-Point Probe Technology Co., Ltd., Guangzhou, China) for measuring the volume resistivity of the composites to characterize their electrical conductivity; a super-depth-of-field microscope (Keyence, Osaka, Japan) for observing the cross-sectional microstructure, SA dispersion, and carbon layer structure of the composites; an electronic universal testing machine (WDW-10A, Jinan Zhonglusi Testing Machine Co., Ltd., Jinan, China) for measuring tensile strength and elongation at break in accordance with GB/T 1040-2006 [44]; a Shore hardness tester (LX-A, Shanghai Liuling Instrument Co., Ltd., Shanghai, China) for measuring the Shore A hardness of the composites in accordance with GB/T 2411-2008 [45]; and a thermogravimetric analyzer (Q5000IR, TA Instruments, New Castle, DE, USA) for evaluating thermal stability under a nitrogen atmosphere, with a heating rate of 10 °C/min, within the temperature range of 30–600 °C.

### 3.3. Sample Preparation

Raw Material Pretreatment: Place the TPU pellets into a vacuum drying oven and dry them at 80 °C for 12 h to remove moisture; the purchased finished product SA shall be subjected to additional screening using a 200-mesh sieve to ensure uniform particle size distribution, followed by drying at 120 °C for 6 h to remove adsorbed moisture.

Batching and Mixing: Based on preliminary research, when the SA content exceeds 15%, agglomeration becomes severe, making it difficult to mold the extruded material using injection molding. So, in accordance with the formulation presented in Table 3, TPU and SA were stirred at high speed in a high-speed mixer for 5 min at a rotational speed of 1500 r/min, ensuring the preliminary, uniform dispersion of the SA.

Melt Blending and Granulation: The mixed raw materials were fed into a TE-35 coaxial twin-screw extruder (screw diameter: 35.6 mm; L/D ratio: 40:1) for melt mixing. The temperature profile from the hopper to the extrusion zone was set at 160 °C, 170 °C, 180 °C, 185 °C, 180 °C, and 175 °C, respectively; the screw speed was 300 rpm, and the feeding rate was 20 kg/h. The extruded material was subsequently water-cooled and pelletized using a pelletizer to obtain the composite masterbatch. The masterbatch was then dried at 100 °C for 4 h to remove any moisture adsorbed during the processing stage.

Injection Molding of Specimens: The dried composite masterbatch was injection-molded into standard specimens using an injection molding machine, with a molding temperature of 200 °C, a mold temperature of 40 °C, an injection pressure of 60 MPa, an injection speed of 30 mm/s, a holding pressure of 40 MPa, and a holding time of 30 s. Tensile specimens (Type 5 dumbbell-shaped, 1.0 mm thick), combustion specimens (100 mm × 100 mm × 3 mm), and oxygen index specimens (130 mm × 6.5 mm × 3.2 mm) were prepared.

### 3.4. Performance Testing and Characterization Methods

#### 3.4.1. Thermal Stability Test (TGA)

The measurement was performed using a TG 209 thermogravimetric analyzer (NETZSCH-Gerätebau GmbH, Selb, Germany) in accordance with the ISO 11358 standard [46] under the following conditions: a nitrogen atmosphere with a flow rate of 40 mL/min and a heating rate of 10 °C/min over a temperature range extending from room temperature to 600 °C. The thermal decomposition curves of the samples were recorded to analyze the initial decomposition temperature (T_5%_, the temperature at which 5% mass loss occurs), the temperature of maximum decomposition rate (T_max_), and the residual carbon content at 600 °C.

#### 3.4.2. Infrared Spectroscopy Characterization (FT-IR)

A Nicolet (Mountain, WI, USA) iS50 Fourier Transform Infrared (FTIR) spectrometer was employed, covering a spectral range of 400–4000 cm^−1^ with a resolution of 4 cm^−1^ and 32 scans. The sample was mixed and ground with KBr at a mass ratio of 1:100 and then pressed into a pellet to analyze the interactions between SA and TPU molecules, as well as the changes in chemical structure during thermal decomposition [47,48]. In the data analysis of infrared spectroscopy, the formula for processing raw data of the temperature axis is defined as follows: the temperature (*T*) equals the set starting temperature (*T_S_*) plus the quotient of the difference between the actual termination temperature (*T_E_*) and the starting temperature (*T_S_*) divided by the experimentally set duration (*S_V_*, in minutes), multiplied by the time corresponding to each data point (*S_E_*), and then divided by the experimentally set duration (*S_V_*, in minutes).

(1) $$T=T_{S}+\frac{\left(T_{E}-T_{S}\right)}{S_{V}}\times \frac{S_{E}}{S_{V}}$$

#### 3.4.3. Mechanical Property Testing

In accordance with the standard GB/T 528-2009 [49], “Vulcanized rubber or thermoplastic rubbers—Determination of tensile stress–strain properties”, tensile tests were conducted using an electronic universal testing machine at a tensile speed of 500 mm/min. Each sample was tested five times to obtain an average value, and parameters such as tensile strength and elongation at break were recorded. Here, strain (*S_L_*) is defined as the displacement *D* during tensile testing divided by the sample’s original parallel length *L*, while stress (*S_P_*) is calculated as the applied force *N* during tensile testing divided by the sample’s original width *W*.

(2) $$S_{L}=\varepsilon =\frac{D}{L}$$

(3) $$S_{P}=\frac{N}{W}$$

#### 3.4.4. Combustion Performance Testing

Cone Calorimeter Testing: In accordance with the ISO 5660-1 standard [50], a cone calorimeter was utilized with a radiant heat flux of 50 kW/m^2^. Test specimens measuring 100 mm × 100 mm × 3 mm were wrapped in aluminum foil and positioned horizontally. Key combustion parameters—including heat release rate (HRR), peak heat release rate (HRR), total heat release (THR), smoke production rate (SPR), and total smoke production (TSR)—were measured and recorded [51,52].

Limiting Oxygen Index (LOI) Test: Conducted per ASTM D2863 [53] with specimen dimensions of 130 mm × 6.5 mm × 3.2 mm. LOI ≥ 27% is defined as self-extinguishing, and LOI ≥ 30% as flame-retardant material. The combined metrics of LOI ≥ 30% and pHRR ≤ 350 kW/m^2^ are established as the combustibility determination criteria.

Observation of Char Layer Morphology: Following combustion testing, the samples were sputter-coated with gold. A super-depth-of-field microscope was then employed to observe the microscopic morphology of the char layer’s surface and cross-section, thereby analyzing its compactness and integrity.

## 4. Conclusions

In this study, SA/TPU composite systems with varying contents of SA were prepared via melt blending. The effects of SA on the thermal stability, mechanical properties, and combustion behavior of TPU were systematically investigated, and its flame-retardant mechanism was elucidated. The main conclusions are as follows:

SA slightly reduces both the maximum thermal decomposition temperature and the peak value of TPU; by forming a dense carbon layer in advance, the concurrently generated antimony-based oxide fills and repairs the microstructural defects within this carbon layer, thereby enhancing the physical–thermal barrier capability of the residual carbon and effectively preventing the conduction of heat into the substrate. This approach leverages a dual role—both catalytic promotion and barrier inhibition—thereby effectively preventing the occurrence of subsequent concentrated heat release phenomena. As a result, the residual carbon content at 600 °C increased significantly from 9.84% to 15.76%, representing a rise of 60%.

As the SA content increases, the mechanical properties of the material decline. A moderate SA addition level (content ≤ 5%) has a relatively minor impact on the mechanical properties of TPU; for instance, the TPU-SA5 sample exhibits a tensile strength of 1.7 MPa and maintains a fracture elongation of 30%, indicating an improvement in its mechanical properties due to a certain degree of reinforcement effect. However, when the SA addition level is high (≥7%), the mechanical properties decrease significantly owing to particle agglomeration.

SA effectively enhances the flame retardancy and smoke suppression properties of TPU; the LOI value of the TPU-SA10 group reached 32.8%, and it achieved a V-0 rating in the vertical burning test. Furthermore, the pHRR and THR were reduced by 50% and 40%, respectively, compared to pure TPU, resulting in a significant reduction in fire hazard.

The flame retardancy mechanism of SA in TPU involves a synergistic effect between the condensed phase and the gas phase: in the condensed phase, it catalyzes the formation of a dense char layer that acts as a barrier against heat and oxygen; in the gas phase, it captures active free radicals to terminate chain reactions.

This study offers the first systematic evaluation of sodium antimonate as a sole flame retardant for TPU, defining a critical loading threshold (≤5 wt%) that balances fire safety and mechanical integrity. The dual-phase mechanism—condensed-phase charring and gas-phase radical quenching—is rigorously validated. Compared to halogenated or high-phosphorus systems, SA provides halogen-free, low-toxicity efficiency while preserving TPU’s microstructure. Practical guidelines are provided for fire-safe applications (e.g., battery enclosures, flexible electronics). Future work should address particle surface modification, synergistic hybrids, and long-term durability to enable sustainable industrial deployment.

## Figures and Tables

**Table 1:** Temperature and residual carbon content scale for each quality loss stage.

Sample	T_5%_ (°C)	T_50%_ (°C)	T_max_ (°C)	Residue (%)
TPU-SA0	301.84	389.18	419.52	9.84
TPU-SA1	298.23	345.13	380.17	11.23
TPU-SA3	295.68	336.31	330.89	12.56
TPU-SA5	293.57	340.11	335.34	13.12
TPU-SA7	292.19	338.53	340.63	13.89
TPU-SA10	290.80	341.24	345.58	15.76

**Table 2:** The parameters of the material’s mechanical properties.

Sample	Tensile Strength (MPa)	Elongation at Break (%)	Elastic Modulus (MPa)	Toughness (MJ/m^3^)
TPU-SA0	5.6 ± 0.3	280 ± 10%	2.25 ± 0.09	11.4 ± 0.5
TPU-SA1	3.6 ± 0.2	70 ± 10%	5.84 ± 0.23	1.71 ± 0.07
TPU-SA3	2.1 ± 0.1	20 ± 6%	17.39 ± 1.04	0.21 ± 0.01
TPU-SA5	1.7 ± 0.1	30 ± 6%	13.92 ± 0.84	0.25 ± 0.02
TPU-SA7	1.4 ± 0.1	15 ± 6%	14.19 ± 0.85	0.11 ± 0.01
TPU-SA10	1.3 ± 0.1	10 ± 6%	11.17 ± 0.67	0.09 ± 0.01

**Table 3:** Formulation design of the SA/TPU composite system.

Sample	TPU Mass Fraction (%)	SA Mass Fraction (%)	Remark
TPU-SA0	100	0	Pure TPU Control Group
TPU-SA1	99	1	Low-Addition Group
TPU-SA3	97	3	Low-to-Medium Addition Group
TPU-SA5	95	5	Medium-Dosage Group
TPU-SA7	93	7	Medium-to-High-Addition Group
TPU-SA10	90	10	High-Addition Group

## Data Availability

The data that support the findings of this study are available from the corresponding author upon reasonable request.
